# Antitrypanosomal Activity of Novel Benzaldehyde-Thiosemicarbazone Derivatives from Kaurenoic Acid ^†^

**DOI:** 10.3390/molecules16021166

**Published:** 2011-01-26

**Authors:** Shirani K. Haraguchi, Adriano A. Silva, Gentil J. Vidotti, Phercyles V. dos Santos, Francielle P. Garcia, Raissa B. Pedroso, Celso V. Nakamura, Cecília M. A. de Oliveira, Cleuza C. da Silva

**Affiliations:** 1 Departamento de Química, Universidade Estadual de Maringá, Av. Colombo, 5790, 87020-900, Maringá, PR, Brazil; E-Mails; shi_haraguchi@hotmail.com (S.K.H.), adriano_a_silva@hotmail.com (A.A.S.); 2 Departamento de Análises Clínicas, Universidade Estadual de Maringá, Av. Colombo, 5790, 87020-900, Maringá, PR, Brazil; E-Mails: phercyles@hotmail.com (P.V.S.); fran_cielle_pg@hotmail.com (F.P.G.); raissap@hotmail.com (R.B.P.); cvnakamura@uem.br (C.V.N.); 3 Instituto de Química, Universidade Federal de Goiás, Campus Samambaia, CP 131, 74001-970, Goiânia – GO, Brazil; E-Mail: cecília@quimica.ufg.br (C.M.A.O.)

**Keywords:** kaurenoic acid, thiosemicarbazone, *Trypanosoma cruzi*, Chagas desease

## Abstract

A series of new thiosemicarbazones derived from natural diterpene kaurenoic acid were synthesized and tested against the epimastigote forms of *Trypanosoma cruzi* to evaluate their antitrypanosomal potential. Seven of the synthesized thiosemicarbazones were more active than kaurenoic acid with IC_50_ values between 2-24.0 μM. The *o*-nitro-benzaldehyde-thiosemicarbazone derivative was the most active compound with IC_50_ of 2.0 μM. The results show that the structural modifications accomplished enhanced the antitrypanosomal activity of these compounds. Besides, the thiocyanate, thiosemicarbazide and the *p*- methyl, *p*-methoxy, *p*-dimethylamine, *m*-nitro and *o*-chlorobenzaldehyde-thiosemicarbazone derivatives displayed lower toxicity for LLMCK_2_ cells than kaurenoic acid, exhibing an IC_50_ of 59.5 μM.

## 1. Introduction

Chagas’ disease, caused by the hemoflagellate protozoan *Trypanosoma cruzi* (family Trypanosomatidae, order Kinetoplastida) [1,2], is considered a neglected disease and is a public health problem affecting approximately 18 million people, mainly in Latin America [2,3]. The current treatment is based on two drugs, nifurtimox (4-[(5-nitrofurfurylidene)-amino]-3-methylthio morpholine-1,1-dioxide) and benznidazol (*N*-benzyl-2-nitro-1-imidazoleacetamide), which are highly toxic to mammalian cells [4,5,6] and are not specific to all *T. cruzi* strains. For these reasons the development of more efficient and safer drugs to treat Chagas’ disease is of great importance. 

The kauranes diterpenes are a class of compounds that occur naturally in plants and present several interesting biological activities, such as plant growth regulation and antimicrobial, antiparasitic, insect antifeedant, cytotoxic, antitumoral, anti-HIV, steroidogenic, antifertility, hypotensive, and anti-inflammatory properties [7,8]. Kaurenoic acid, an *ent*-kaurane diterpene that possesses a wide spectrum of bioactivities, such as anti-inflammatory [9], antiproliferative [10], antitrypanosomal [11,12,13], antitumoral [14], antibacterial [15] and antifungal [16] activities, is not commercially available, but is relatively abundant in some species belonging to the *Wedelia*, *Mikania*, *Annona*, *Xylopia* [8], *Acmela* [17] and *Croton* [18,19] genera, enabling their use as natural sources of this diterpene.

The thiosemicarbazones, an important class of synthetic compounds, have a variety of applications due to their wide spectrum of biological activities [20,21], which include antiviral [22], and antitumoral [23,24,25] activities among others as well as parasiticidal activity against *Plasmodium falciparum and Plasmodium berghei* [26], *Trypanosoma cruzi* [27,28,29] and *Trypanosoma brucei rhodesiense* [30], and *Toxoplasma gondii* [31].

Considering the wide range of anti-cancer and antiparasitic activities derived from natural products, and considering that the vast majority of the thiosemicarbazones described in the literature only present structural variations on the imine carbon, our research group began the synthesis of new thiosemicarbazones containing mono- and sesquiterpenic units linked to the terminal nitrogen. 

In previous research, several thiosemicarbazone derivatives of (-)-α-bisabolol exhibited inhibitory effects on the growth of eight cancer cell lines outlining myeloid leukemia (K-562) as especially sensitive to all of the tested compounds. Among the analogues, the methyl-phenyl-ketone derivative was the most active, exhibiting potent antitumoral activity (GI_50_ 0.01 μM) and high selectivity for K-562 (δTGI 505). This derivative also demonstrated high cytotoxicity (IC_50_ 1.55 μM) for K-562 with moderate selectivity (δLC_50_ 38.5 μM) [32]. Additionally, a thiosemicarbazide derivative of camphene displayed activity against *Trichophyton mentagrophytes*, a dermatophyte fungus [33]. In a continuation of our research efforts, we selected kaurenoic acid as a starting point to find new drugs with reduced side effects and greater efficacy in the chemoprophylaxis and chemotherapy of Chagas’ disease, as kaurenoic acid has been previously described as a valuable asset against *T. cruzi*. 

## 2. Results and Discussion

### 2.1. Synthesis

**Scheme 1:**
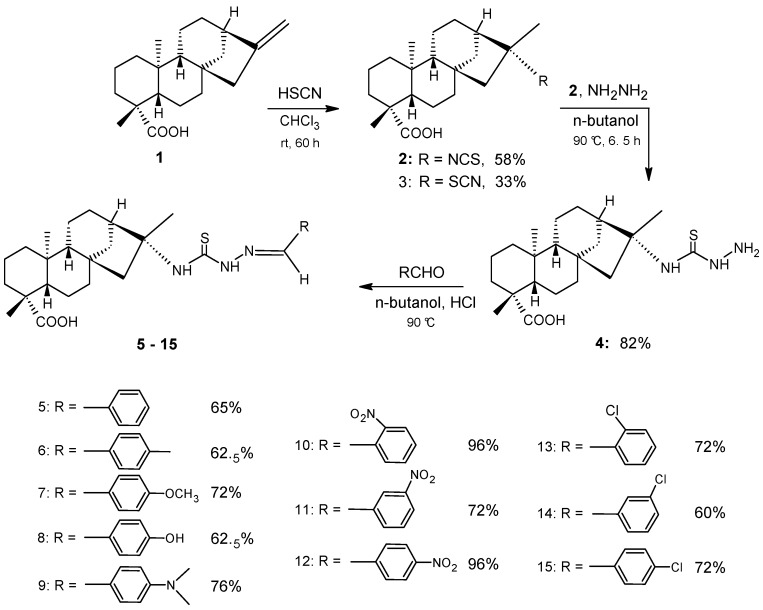
Synthetic route of the preparation of benzaldehyde-thiosemicarbazones derivatives **5-15** of kaurenoic acid (**1**).

The synthetic route for the preparation of benzaldehyde-thiosemicarbazone derivatives of kaurenoic acid is presented in Scheme 1. The kaurenoic acid isothiocyanate and thiocyanate derivatives **2** and **3** were obtained as a mixture from a reaction between kaurenoic acid (**1**) and HSCN performed according to a previously reported procedure [34,35]. The reaction yield was of 58% of **2** and 33% of **3**. The mainly difference between this compounds was observed by the signals moieties in ^13^C-NMR spectrum at δ_c_ 128.6 (NCS) and δ_c_ 113.5 (SCN). In product **2**, the presence of the NCS moiety was also demonstrated by the IR absorption band at 2,125 cm^-1^ and the diterpenic substituent was further characterized by the signals at δ_H_/δ_C_ 1.52 (s, 3H, H-17)/23.6 (C-17), 1.24 (3H, s, H-18)/29.0 (C-18), 185.0 (C-19) and 0.94 (s, 3H, H-20)/15.6 (C-20) in the ^1^H/^13^C-NMR spectrum.

The kaurenoic acid thiosemicarbazide derivative **4** was obtained in 82% yield from the addition reaction of **2** with hydrazine according to a previously reported procedure [32,33]. In product **4**, the presence of the NH moieties were determined by the IR absorption bands at 3,311, 3,198 and 1,620 cm^-1^ (NH_2_) as well as at 2,941 cm^-1^ (NH). This IR information was combined with the signals at δ_H _4.48 (brs, 2H, N-1), 8.40 (brs, 1H, N-2), and 7.45 (s, 1H, N-4) in the ^1^H-NMR spectrum and the signal at δ_C _179.3 (C-3) in the ^13^C-NMR spectrum.

The kaurenoic acid benzaldehyde-thiosemicarbazone derivatives **5-15** were obtained with yields between 60 and 96%, through the condensation of thiosemicarbazide **4** with benzaldehyde and its derivatives, which were substituted at the *para* position with methyl, methoxyl, hydroxyl, and dimethylamine groups and at the *ortho*, *meta*, and *para* positions with nitro and chloro groups according to a previously reported procedure [32]. The benzaldehyde-thiosemicarbazone moiety was characterized by NH signals, in which one was linked to the imine portion between δ_H _11.11-11.67 (s, 1H, N-2), another linked to the terpenic portion between δ_H _7.41-7.63 (s, 1H, N-4), and the last to the imine hydrogen between δ_H _7.93-8.44 (s, 1H, H-1″)/ δ_C_ 121.4-142.5 (C-1’’). In addition, the signals for the hydrogens and carbons of the aromatic system (H-2″ - H-7″ and C-2″ - C-7″) between δ_H _6.72-8.43/δ_C _111.9-160.7 in the ^1^H/^13^C-NMR spectrum were also used for characterization. The remaining NMR data were consistent for the various substituents on the phenyl moiety discussed in the Experimental Section.

### 2.2. Biological assays

The results of the antitrypanosomal (IC_50_) and cytotoxic (CC_50_) assays, as well as their respective selectivity indexes (SI), are shown in Table 1. Through the antitrypanosomal assay evaluation of kaurenoic acid (**1**) and its derivatives **2-15**, it was verified that excluding the thiosemicarbazide **4** and *p*-nitrobenzaldehyde-thiosemicarbazone **12**, all of the derivatives synthesized were more active than kaurenoic acid (**1**). The *o*-nitrobenzaldehyde-thiosemicarbazone derivative **10 **was of particular interest, as it was the most active compound, with an IC_50_ of 2.0 μM. These results indicated that these structural modifications enhanced the antitrypanosomal activity of these compounds when compared to kaurenoic acid in this assay.

**Table 1:** CC_50_ values for LLMCK_2_ cells and IC_50_ values for epimastigote form of *Trypanosoma cruzi* and theirs respective selectivity indexes (SI).

	LLMCK_2_ CC_50_	Epimastigote IC_50_	
	μM ± SD^b^		SI^a^
**1**	59.5 ± 0.1	101.7 ± 0.0	0.6
**2**	52.0 ± 0.3	58.2 ± 0.2	0.9
**3**	103.4 ± 0.7	43.4 ± 5.6	2.4
**4**	71.4 ± 0.7	107.0 ± 13.0	0.7
**5**	31.1 ± 1.0	68.2 ± 11.2	0.5
**6**	122.4± 1.0	23.4 ± 7.7	7.3
**7**	78.2 ± 0.0	16.0 ± 4.4	5.7
**8**	29.5 ± 0.8	18.3 ± 4.0	1.9
**9**	248.5 ± 0.6	79.5 ± 17.9	3.2
**10**	17.1 ± 0.1	2.0 ± 1.1	9.0
**11**	104.8 ± 0.4	19.0 ± 0.0	5.6
**12**	15.2 ± 0.0	116.6 ± 12.8	0.1
**13**	364.2 ± 11.3	39.4 ± 14.3	8.4
**14**	56.2 ± 0.4	23.5 ± 7.0	3.2
**15**	28.7 ± 1.1	14.9 ± 0.3	2.0

^a^ SI: CC_50_ LLMCK_2_ / IC_50_ epimastigote; ^b^ SD: Standard Deviation

The incorporation of HSCN into kaurenoic acid increased the activity of the isothiocyanate and thiocyanatederivatives **2** and **3** by approximately two-fold (58.4 and 43.7 µM, respectively). With the incorporation of the hydrazine on the isothiocyanate **2**, there was a decrease in the activity of the thiosemicarbazide **4** to the same order of activity displayed by kaurenoic acid (**1**) (101.7 µM). Hence, the incorporation of the benzaldehyde increased the activity of the benzaldehyde-thiosemicarbazone derivative **5** to the same order of magnitude as the isothiocyanate derivative **2** (68.2 µM).

The methyl, methoxyl and hydroxyl substituents, which are electron density donors, at the *para* position of the aromatic ring increased the activity of the thiosemicarbazones **6**, **7,** and **8** (23.4, 16.0, and 18.3 µM) approximately three-fold when compared to compound **5. **Conversely, the incorporation of a dimethylamine substituent, also an electron density donor, at the same position decreased the activity of compound **9** to 79.5 µM. 

A decrease in the activity (2.0, 19.0, and 116.6 µM, respectively) was observed for the nitro substituent, a strong electron density withdrawer when it was in the *ortho* (**10**), *meta* (**11**), and *para* (**12**) positions. Contrary to the nitro group, the chloro substituent, a weak electron density withdrawer, increased activity when in the *ortho* (**13**), *meta* (**14**), and *para* (**15**) positions (39.4, 23.5, and 14.9 µM, respectively).

Through the cytotoxicity evaluation, it was observed that *p*-dimethylaminobenzaldehyde-thiosemicarbazone **9** and *o*-chlorobenzaldehyde-thiosemicarbazone **13** presented the smallest toxic effects to the LLMCK_2_ cells (248.5 and 364.2 µM, respectively), and the *p*-nitrobenzaldehyde-thiosemicarbazone **12** and *o*-nitrobenzaldehyde-thiosemicarbazone **10** the biggest (15.2 and 17.1 µM).

From the observed data for the selectivity index (SI), the synthesized compounds **3**, **6**, **7**, **8**, **9**, **10**, **11**, **13**, **14**, and **15 **all had SI values greater than 1.9, which means that this compounds were almost twice or more selective for the pathologic agent than for the host cell. Among this compounds, *o*-nitro (**10**), *o*-chloro (**13), ***p*-methyl- **(6),** p-methoxyl (**7**) and *m*-nitrobenzaldehyde-thiosemicarbazone (**11**) were the most selective, with SI values 9.0, 8.4, 7.3, 5.7 and 5.6, respectively.

These results indicated that electronic effects were not the only factor governing biological activity. However, further investigations are necessary to elucidate the mode of action, and structure-activity correlations must be done to understand the effects involved in activity. With this information in hand, these compounds could then be used as new medicines for combating Chagas’ disease.

## 3. Experimental

### 3.1. General

All melting points were determined using a Microquímica model MQAPF-301 apparatus and are uncorrected. IR spectra were obtained using KBr pellets on an FT-IR BOMEM spectrophotometer. Low resolution mass spectra were recorded by means of a SHIMADZU-CG/MS model QP 2000A spectrometer at 70 eV with a prob for solids. The optical rotations were determined in CHCl_3 _or DMSO as a solvent with a Perkin Elmer polarimeter 343 model at 20 °C. Proton nuclear magnetic resonance (^1^H NMR) spectra were recorded using CDCl_3_ or DMSO-*d_6_* as a solvent, at ambient temperature, on a Varian Mercury plus BB 300 spectrometer (300 MHz) with TMS as an internal standard. The chemical shifts (*δ*) are given in parts per million relative to TMS. arbon-13 nuclear magnetic resonance (^13^C NMR) spectra were recorded at 75.5 MHz with the same internal standard. 

Chromatography was performed on silica gel Merck 230–400 mesh ASTM. 

The solvents were previously treated under reduced pressure and the reagents (Sigma-Aldrich) were used as received.

### 3.2. Plant material

Dried bark of *Croton floribundus* (Euphorbiaceae) was collected in the 2-km area of the PR 323 highway in Maringá, Paraná, Brazil, during 2006 and was identified by comparison with a voucher specimen deposited at the Herbarium of the Universidade Estadual de Maringá under registration code HUEM 8406.

### 3.3. Extraction and isolation of kaurenoic acid *(**1**)*

Dried, powdered material (5.5 kg) was extracted with hexane (1 L) for 12 h using a Soxhlet apparatus. The extract was then concentrated using a rotary evaporator under reduced pressure below 40 °C and then concentrated under vacuum at room temperature. The dried, crude hexane extract was chromatographed on a silica gel column using a hexane–dichloromethane gradient solvent system. The fractions that contained kaurenoic acid were gathered and recrystallized from cold methanol. This procedure resulted in 27.0 g of pure kaurenoic acid (**1**, *ent*-kaur-16-en-19-oic acid**)** as a white powder. m.p. 177 °C, [α]^20^_D_: -105.20 deg dm^-1 ^g^-1^ cm^3^ (*c* 0.102, CHCl_3_). The spectroscopic data were consistent with the literature [13,16].

### 3.4. General procedure for the preparation of isothiocyanate ***2***

*Isothiocyanic acid* [34,35]: a slurry of powdered KSCN (7.92 mmol) in CHCl_3_ (20 mL) was triturated with KHSO_4_ (7.92 mmol) in a mortar for 5 min. The HSCN chloroform solution was decanted, an additional 10 mL of CHCl_3_ was added to the solid mixture, and the solution was again decanted. The combined organic solutions totaled 30 mL in volume. The isolated kaurenoic acid (**1**) (0.66 mmol) and the HSCN/CHCl_3_ solution (30 mL) were stirred at room temperature for 60 h. The reaction was monitored by TLC using kaurenoic acid (**1**) as the reference standard. After filtration, the solvent was removed under reduced pressure. The residue was purified by chromatography on a silica gel column eluted with hexane/ethyl acetate 5-15%, giving isothiocyanate (**2**) (58% yield) and the more polar thiocyanate (**3**) (33% yield).

*ent-Kauren-16α-isothiocyane-16β-methyl-19-oic acid* (**2**). White amorphous powder; m.p. 174 °C; [α]^20^_D_: -106.29 deg dm^-1 ^g^-1^ cm^3^ (*c* 0.097, CHCl_3_); IR (KBr) υ_max_/cm^-1^: 2,125 (NCS), 1,693 (C=O); EI-MS m/z: 361 (M^+•^); ^1^H-NMR δ_H_ (300 MHz, CDCl_3_): 0.70-0.82 (m, 1H, H-1) and 1.74-1.86 (m, 1H, H-1), 1.34-1.50 (m, 1H, H-2), and 1.83-1.90 (m, 1H, H-2), 0.92-1.06 (m, 1H, H-3) and 2.16 (d, 1H, *J* = 15.6 Hz, H-3), 0.96-1.10 (m, 1H, H-5), 1.70-1.89 (m, 2H, H-6), 1.30-1.45 (m, 1H, H-7) and 1.60-1.70 (m, 1H, H-7), 0.90-1.02 (m, 1H, H-9), 1.50-1.64 (m, 2H, H-11), 1.48-1.60 (m, 1H, H-12) and 2.05 (dl, 1H, *J* = 12.3 Hz, H-12), 2.18 (brs, 1H, H-13), 1.42-1.64 (m, 2H, H-14), 1.50-1.62 (m, 1H, H-15) and 1.82-1.95 (m, 1H, H-15), 1.52 (s, 3H, H-17), 1.24 (s, 3H, H-18), 0.94 (s, 3H, H-20); ^13^C-NMR δ_C_ (75.5 MHz, CDCl_3_): 40.7 (C-1), 19.1 (C-2), 37.8 (C-3), 45.5 (C-4), 56.8 (C-5), 22.0 (C-6), 41.6, (C-7), 43.8 (C-8), 55.8 (C-9), 39.8 (C-10), 18.3 (C-11), 38.4 (C-12), 48.5 (C-13), 26.4 (C-14), 56.5 (C-15), 69.2 (C-16), 23.6 (C-17), 29.0 (C-18), 185.0 (C-19), 15.6 (C-20), 128.6 (C-21).

*ent-Kauren-16α-thiocyane-16β-methyl-19-oic acid* (**3**). White amorphous powder; m.p. 165 °C; [α]^20^_D_: -72.45 deg dm^-1 ^g^-1^ cm^3^ (*c* 0.102, CHCl_3_); IR (KBr) υ_max_/cm^-1^: 2,144 (SCN), 1,693 (C=O); EI-MS m/z: 361 (M^+•^); ^1^H-NMR δ_H_ (300 MHz, CDCl_3_): 0.70-0.80 (m, 1H, H-1) and 1.78-1.90 (m, 1H, H-1), 1.36-1.52 (m, 2H, H-2), 0.90-1.00 (m, 1H, H-3) and 2.16 (d, 1H, *J* = 12.6 Hz, H-3), 1.00-1.08 (m, 1H, H-5), 1.78-1.88 (m, 2H, H-6), 1.34-1.47 (m, 1H, H-7) and 1.54-1.64 (m, 1H, H-7), 0.94-1.04 (m, 1H, H-9), 1.56-1.67 (m, 2H, H-11), 1.66-1.76 (m, 1H, H-12) and 2.06 (d, 1H, *J* = 12.3 Hz, H-12), 2.27 (m, 1H, H-13), 1.60-1.73 (m, 2H, H-14), 1.64 (s, 2H, H-15), 1.80 (s, 3H, H-17), 1.24 (s, 3H, H-18), 0.95 (s, 3H, H-20); ^13^C-NMR δ_C_ (75.5 MHz, CDCl_3_): 40.7 (C-1), 19.1 (C-2), 37.8 (C-3), 46.3 (C-4), 56.8 (C-5), 22.0 (C-6), 41.8, (C-7), 43.9 (C-8), 55.9 (C-9), 39.8 (C-10), 18.6 (C-11), 38.9 (C-12), 47.3 (C-13), 27.6 (C-14), 54.5 (C-15), 63.9 (C-16), 25.6 (C-17), 29.1 (C-18), 184.7 (C-19), 15.5 (C-20), 113.5 (C-21).

### 3.5. General procedure for the preparation of thiosemicarbazide ***4***

Isothiocyanate **2** (0.55 mmol), dissolved in *n*-butanol (50 mL) and hydrazine (1.10 mmol), was stirred at 90 °C for 6.5 h. The reaction was monitored by TLC using isothiocyanate **2** as the reference standard. The mixture was then partitioned in *n*-butanol/distilled water, and the butanol phase was washed with CHCl_3_ to afford the thiosemicarbazide **4** in 82% yield.

*N4-[ent-Kauren-16β-methyl-19-oic acid]-thiosemicarbazide* (**4**). White amorphous powder; m.p. 131 °C; [α]^20^_D_: -66.67 deg dm^-1 ^g^-1^ cm^3^ (*c* 0.024, DMSO); IR (KBr) υ_max_/cm^-1^: 3,311, 3,198 and 1,620 (NH_2_), 2,941 (NH), 1,251 (C=S); EI-MS m/z: 393 (M^+•^); ^1^H-NMR δ_H_ (300 MHz, DMSO-*d_6_*): 4.48 (brs, 2H, N-1), 8.40 (brs, 1H, N-2), 7.45 (s, 1H, N-4), 0.62-0.74 (m, 1H, H-1’) and 1.62-1.76 (m, 1H, H-1’), 1.13-1.28 (m, 2H, H-2’), 0.68-0.80 (m, 1H, H-3’) and 2.01 (d, 1H, *J =* 12.0 Hz, H-3’), 0.74-0.86 (m, 1H, H-5’), 1.60-1.74 (m, 1H, H-6’) and 1.74-1.86 (m, 1H, m, H-6’), 1.22-1.40 (m, 2H, H-7’), 0.86-0.94 (m, 1H, H-9’), 1.40-1.54 (m, 2H, H-11’), 1.14-1.26 (m, 1H, H-12’) and 1.78-1.90 (m, 1H, H-12’), 2.33 (brs, 1H, H-13’), 1.38-1.52 (m, 1H, H-14’) and 1.52-1.63 (m, 1H, H-14’), 1.40-1.52 (m, 1H, H-15’) and 2.14 (d, 2H, *J =* 14.7 Hz, H-15’), 1.62 (s, 3H, H-17’), 0.98 (s, 3H, H-18’), 0.92 (s, 3H, H-20’); ^13^C-NMR δ_C_ (75.5 MHz, DMSO-*d_6_*): 179.6 (C-3), 41.0 (C-1’), 19.5 (C-2’), 37.5 (C-3’), 44.7 (C-4’), 56.8 (C-5’), 22.5 (C-6’), 42.6, (C-7’), 43.3 (C-8’), 55.7 (C-9’), 39.8 (C-10’), 18.2 (C-11’), 39.1 (C-12’), 46.2 (C-13’), 26.3 (C-14’), 56.7 (C-15’), 62.2 (C-16’), 21.1 (C-17’), 29.8 (C-18’), 181.0 (C-19’), 15.7 (C-20’).

### 3.6. General procedure for the synthesis of benzaldehyde-thiosemicarbazones ***5-15***

Thiosemicarbazide **4** (0.50 mmol) was dissolved in *n*-butanol (50 mL) and then treated with 0.2% hydrochloric acid (50 µL) and benzaldehyde derivatives (0.50 mmol); it was then stirred at 90 °C for a period of time specific to each synthesized compound. The reactions were monitored by TLC using thiosemicarbazide **4** as the reference standard. This mixture was partitioned between *n*-butanol and distilled water, and the solvent was removed from the butanol phase under reduced pressure. The benzaldehyde-thiosemicarbazones **5-15** were then recrystallized from acetone.

*N1-(E)-Phenyl-N4-[ent-kauren-16β-methyl-19-oic acid]-thiosemicarbazone* (**5**). Reaction time: 6 h, 65% yield; white amorphous powder; m.p. 202 °C; [α]^20^_D_: -15.38 deg dm^-1 ^g^-1^ cm^3^ (*c* 0.026, DMSO); IR (KBr) υ_max_/cm^-1^: 3,341 (NH), 1,694 (C=O), 1,530-1,490, 755 and 690 (C=C aromatic), 1,241 (C=S); EI-MS m/z: 481 (M^+•^); ^1^H-NMR δ_H_ (300 MHz, DMSO-*d_6_*): 11.38 (s, 1H, N-2), 7.51 (s, 1H, N-4), 0.72-0.80 (m, 1H, H-1’) and 1.66-1.80 (m, 1H, H-1’), 1.22-1.34 (m, 2H, H-2’), 0.82-0.92 (m, 1H, H-3’) and 1.99 (d, 1H, *J* = 12.3 Hz, H-3’), 0.99 (m, 2H, H-5’ and H-9’), 1.60-1.72 (m, 2H, H-6’), 1.30-1.42 (m, 2H, H-7’), 1.45-1.58 (m, 2H, H-11’), 1.24-1.32 (m, 1H, H-12’) and 1.89 (d, 1H, *J =* 11.1 Hz, H-12’), 2.43 (brs, 1H, H-13’), 1.42-1.52 (m, 1H, H-14’) and 1.58-1.66 (m, 1H, H-14’), 1.44-1.52 (m, 1H, H-15’) and 2.27 (d, 1H, *J =* 15.0 Hz, H-15’), 1.69 (s, 3H, H-17’), 1.07 (s, 3H, H-18’), 11.95 (brs, 1H, H-19’), 0.88 (s, 3H, H-20’), 8.05 (s, 1H, H-1’’), 7.64 (m, 2H, H-3’’ and H-7’’), 7.34-7.48 (m, 3H, H-4’’, H-5’’ and H-6’’); ^13^C-NMR δ_C_ (75.5 MHz, DMSO-*d_6_*): 175.1 (C-3), 40.2 (C-1’), 18.8 (C-2’), 37.6 (C-3’), 44.6 (C-4’), 55.8 (C-5’), 21.9 (C-6’), 42.1, (C-7’), 42.8 (C-8’), 55.3 (C-9’), 39.1 (C-10’), 18.1 (C-11’), 37.6 (C-12’), 46.5 (C-13’), 26.1 (C-14’), 55.7 (C-15’), 63.2 (C-16’), 20.5 (C-17’), 28.6 (C-18’), 178.6 (C-19’), 15.2 (C-20’), 141.4 (C-1’’), 134.0 (C-2’’), 127.0 (C-3’’ and C-7’’), 128.9 (C-4’’, C-5’’ and C-6’’).

*N1-(E)-[4-Methylphenyl]-N4-[ent-kauren-16β-methyl-19-oic acid]-thiosemicarbazone* (**6**). Reaction time: 2 h, 62.5% yield; white amorphous powder; m.p. 223 °C; [α]^20^_D_: -12.00 deg dm^-1 ^g^-1^ cm^3^ (*c* 0.025, DMSO); IR (KBr) υ_max_/cm^-1^: 3,336 (NH), 1,696 (C=O), 1,539, 1,514, 814 (C=C aromatic), 1,245 (C=S); EI-MS m/z: 495 (M^+•^); ^1^H-NMR δ_H_ (300 MHz, DMSO-*d_6_*): 11.32 (s, 1H, N-2), 7.50 (s, 1H, N-4), 0.72-0.80 and 1.70-1.82 (m, 2H, H-1’), 1.22-1.34 (m, 2H, H-2’), 0.86-0.96 (m, 1H, H-3’) and 2.00 (d, 1H, *J =* 12.9 Hz, H-3’), 1.00 (m, 2H, H-5’ and H-9’), 1.58-1.76 (m, 2H, H-6’), 1.30-1.44 (m, 2H, H-7’), 1.46-1.58 (m, 2H, H-11’), 1.22-1.34 (m, 1H, H-12’) and 1.91 (d, 1H, *J =* 11.7 Hz, H-12’), 2.44 (brs, 1H, H-13’), 1.42-1.55 (m,1H, H-14’) and 1.58-1.66 (m,1H, H-14’), 1.46-1.58 (m,1H, H-15’) and 2.38 (d, 1H, *J* = 5.0 Hz, H-15’), 1.70 (s, 3H, H-17’), 1.09 (s, 3H, H-18’), 11.96 (brs, 1H, H-19’), 0.89 (s, 3H, H-20’), 8.02 (s, 1H, H-1’’), 7.55 (d, 2H, *J* = 8.0 Hz, H-3’’ and H-7’’), 7,23 (d, 2H, *J* = 8.0 Hz, H-4’’ and H-6’’), 2.32 (s, 3H, H-8’’); ^13^C-NMR δ_C_ (75.5 MHz, DMSO-*d_6_*): 175.0 (C-3), 40.1 (C-1’), 18.8 (C-2’), 37.6 (C-3’), 44.6 (C-4’), 55.8 (C-5’), 21.9 (C-6’), 42.0, (C-7’), 42.8 (C-8’), 55.2 (C-9’), 39.1 (C-10’), 18.1 (C-11’), 37.6 (C-12’), 46.4 (C-13’), 26.0 (C-14’), 55.8 (C-15’), 63.1 (C-16’), 20.5 (C-17’), 28.6 (C-18’), 178.6 (C-19’), 15.2 (C-20’), 141.6 (C-1’’), 131.2 (C-2’’), 127.0 (C-3’’ and C-7’’), 129.5 (C-4’’ and C-6’’), 139.8 (C-5’’), 21.1 (C-8’’).

*N1-(E)-[4-Methoxyphenyl]-N4-[ent-kauren-16β-methyl-19-oic acid]-thiosemicarbazone* (**7**). Reaction time: 2 h, 72% yield; light yellow amorphous powder; m.p. 214.5 °C; [α]^20^_D_: -6.80 deg dm^-1 ^g^-1^ cm^3^ (*c* 0.025, DMSO); IR (KBr) υ_max_/cm^-1^: 3,333 (NH), 1,697 (C=O), 1,539, 1,511, 830 (C=C aromatic), 1,249 (C=S and C-O-C); EI-MS m/z: 511 (M^+•^); ^1^H-NMR δ_H_ (300 MHz, DMSO-*d_6_*): 11.26 (s, 1H, N-2), 7.48 (s, 1H, N-4), 0.68-0.84 (m, 1H, H-1’) and 1.70-1.82 (m, 1H, H-1’), 1.24-1.37 (m, 2H, H-2’), 0.86-0.92 (m, 1H, H-3’) and 2.00 (d, 2H, *J =* 13.2 Hz, H-3’), 1.01 (m, 2H, H-5’ and H-9’), 1.60-1.76 (m, 2H, H-6’), 1.32-1.44 (m, 2H, H-7’), 1.45-1.65 (m, 2H, H-11’), 1.20-1.32 (m, 1H, H-12’) and 1.91 (d, 1H, *J* = 11.7 Hz, H-12’), 2.45 (brs, 1H, H-13’), 1.40-1.54 (m, 1H, H-14’) and 1.58-1.68 (m, 1H, H-14’), 1.41-1.59 (m, 1H, H-15’) and 2.27 (d, 1H, *J =* 15.0 Hz, H-15’), 1.70 (s, 3H, H-17’), 1.09 (s, 3H, H-18’), 11.96 (brs, 1H, H-19’), 0.90 (s, 3H, H-20’), 8.00 (s, 1H, H-1’’), 7.60 (d, 2H, *J* = 8.7 Hz, H-3’’ and H-7’’), 6.98 (d, 2H, *J =* 8.7 Hz, H-4’’ and H-6’’), 3.79 (s, 3H, H-8’’); ^13^C-NMR δ_C_ (75.5 MHz, DMSO-*d_6_*): 174.8 (C-3), 40.1 (C-1’), 18.8 (C-2’), 37.6 (C-3’ e C-12’), 44.6 (C-4’), 55.8 (C-5’), 21.9 (C-6’), 42.1, (C-7’), 42.8 (C-8’), 55.3 (C-9’), 39.1 (C-10’), 18.1 (C-11’), 46.5 (C-13’), 26.1 (C-14’), 55.8 (C-15’), 63.1 (C-16’), 20.6 (C-17’), 28.6 (C-18’), 178.6 (C-19’), 15.2 (C-20’), 141.5 (C-1’’), 126.5 (C-2’’), 128.6 (C-3’’ and C-7’’), 114.4 (C-4’’ and C-6’’), 160.7 (C-5’’), 55.3 (C-8’’).

*N1-(E)-[4-Hydroxyphenyl]-N4-[ent-kauren-16β-methyl-19-oic acid]-thiosemicarbazone* (**8**). Reaction time: 4 h, 62.5% yield; yellow amorphous powder; m.p. 191.5 °C; [α]^20^_D_: -6.15 deg dm^-1 ^g^-1^ cm^3^ (*c* 0.026, DMSO); IR (KBr) υ_max_/cm^-1^: 3,334 (NH), 1,694 (C=O), 1,538, 1,514, 834 (C=C aromatic), 1,230 (C=S), 1,165 (C-O); EI-MS m/z: 497 (M^+•^); ^1^H-NMR δ_H_ (300 MHz, DMSO-*d_6_*): 11.18 (s, 1H, N-2), 7.44 (s, 1H, N-4), 0.70-0.80 (m, 1H, H-1’) and 1.68-1.80 (m, 1H, H-1’), 1.24-1.37 (m, 2H, H-2’), 0.88-0.98 (m,1H, H-3’) and 2.09 (d, 1H, *J* = 12.6 Hz, H-3’), 1.00 (m, 2H, H-5’ and H-9’), 1.56-1.74 (m, 2H, H-6’), 1.30-1.42 (m, 2H, H-7’), 1.42-1.58 (m, 2H, H-11’), 1.20-1.32 (m, 1H, H-12’) and 1.91 (d, 1H, *J =* 11.7 Hz, H-12’), 2.43 (m, 1H, H-13’), 1.40-1.66 (m, 2H, H-14’), 1.44-1.58 (m, 1H, H-15’) and 2.37 (d, 1H, *J* = 15.0 Hz, H-15’), 1.70 (s, 3H, H-17’), 1.09 (s, 3H, H-18’), 11.96 (brs, 1H, H-19’), 0.89 (s, 3H, H-20’), 7.96 (s,1H, H-1’’), 7.48 (d, 2H, *J =* 8.5 Hz, H-3’’ and H-7’’), 6.80 (d, 2H, *J* = 8.5 Hz, H-4’’ and H-6’’), 9.93 (s,1H, H-8’’); ^13^C-NMR δ_C_ (75.5 MHz, DMSO-*d_6_*): 174.7 (C-3), 40.2 (C-1’), 18.8 (C-2’), 37.6 (C-3’ e C-12’), 44.6 (C-4’), 55.8 (C-5’), 21.9 (C-6’), 42.1, (C-7’), 42.8 (C-8’), 55.3 (C-9’), 39.1 (C-10’), 18.1 (C-11’), 46.5 (C-13’), 26.1 (C-14’), 55.8 (C-15’), 63.0 (C-16’), 20.6 (C-17’), 28.6 (C-18’), 178.6 (C-19’), 15.2 (C-20’), 142.0 (C-1’’), 124.9 (C-2’’), 128.7 (C-3’’ and C-7’’), 115.8 (C-4’’ and C-6’’), 159.4 (C-5’’).

*N1-(E)-[4-Dimethylaminophenyl]-N4-[ent-kauren-16β-methyl-19-oic acid]-thiosemicarbazone* (**9**). Reaction time: 3 h, 76% yield; yellow amorphous powder; m.p. 231 °C; [α]^20^_D_: -6.54 deg dm^-1 ^g^-1^ cm^3^ (*c* 0.026, DMSO); IR (KBr) υ_max_/cm^-1^: 3,323 (NH), 1,686 (C=O), 1,530-1,495, 813 (C=C aromatic), 1,365 (C-N), 1,251 (C=S); EI-MS m/z: 524 (M^+•^); ^1^H-NMR δ_H_ (300 MHz, DMSO-*d_6_*): 11.11 (s, 1H, N-2), 7.44 (s, 1H, N-4), 0.70-0.88 (m, 1H, H-1’) and 1.70-1.80 (m, 1H, H-1’), 1.23-1.36 (m, 2H, H-2’), 0.92-1.00 (m, 1H, H-3’) and 2.00 (d, 1H, *J* = 12.9 Hz, H-3’), 1.00 (m, 2H, H-5’ and H-9’), 1.60-1.74 (m, 2H, H-6’), 1.30-1.46 (m, 2H, H-7’), 1.43-1.62 (m, 2H, H-11’), 1.22-1.30 (m, 1H, H-12’) and 1.90 (d, 1H, *J* = 11.4 Hz, H-12’), 2.44 (brs,1H, H- 13’), 1.41-1.55 (m,1H, H-14’) and 1.57-1.68 (m,1H, H-14’), 1.45-1.58 (m,1H, H-15’) and 2.32 (d, 1H, *J* = 15.0 Hz, H-15’), 1.70 (s, 3H, H-17’), 1.09 (s, 3H, H-18’), 11.96 (brs,1H, H-19’), 0.89 (s, 3H, H-20’), 7.93 (s, 1H, H-1’’), 7.45 (d, 2H, *J* = 9.0 Hz, H-3’’ and H-7’’), 6.72 (d, 2H, *J=* 9.0 Hz, H-4’’ and H-6’’), 2.96 (s, 6H, H-8’’ and H-9’’); ^13^C-NMR δ_C_ (75.5 MHz, DMSO-*d_6_*): 174.4 (C-3), 40.2 (C-1’), 18.8 (C-2’), 37.6 (C-3’ e C-12’), 44.6 (C-4’), 55.8 (C-5’), 21.9 (C-6’), 42.1, (C-7’), 42.8 (C-8’), 55.3 (C-9’), 39.1 (C-10’), 18.1 (C-11’), 46.4 (C-13’), 26.1 (C-14’), 56.0 (C-15’), 62.9 (C-16’), 20.7 (C-17’), 28.6 (C-18’), 178.6 (C-19’), 15.2 (C-20’), 142.5 (C-1’’), 121.2 (C-2’’), 128.3 (C-3’’ and C-7’’), 111.9 (C-4’’ and C-6’’), 151.4 (C-5’’), 38.0 (C-9’’ and C-10’’).

*N1-(E)-[2-Nitrophenyl]-N4-[ent-kauren-16β-methyl-19-oic acid]-thiosemicarbazone*** (10)**. 12 h, 96% yield; yellow amorphous powder; m.p. 211 °C; [α]^20^_D_: -16.80 deg dm^-1 ^g^-1^ cm^3^ (*c* 0.025, DMSO); IR (KBr) υ_max_/cm^-1^: 3,339 (NH), 1,697 (C=O), 1,530-1,500, 784 (C=C aromatic), 1,343 (N-O), 1,246 (C=S); EI-MS m/z: 526 (M^+•^); ^1^H-NMR δ_H_ (300 MHz, DMSO-*d_6_*): 11.67 (s,1H, N-2), 7.41 (s,1H, N-4), 0.70-0.80 (m, 1H, H-1’) and 1.72-1.80 (m, 1H, H-1’), 1.24-1.36 (m, 2H, H-2’), 0.82-0.94 (m, 1H, H-3’) and 2.00 (d, 1H, *J* = 12.9 Hz, H-3’), 1.00 (m, 2H, H-5’ and H-9’), 1.58-1.72 (m, 2H, H-6’), 1.30-1.48 (m, 2H, H-7’), 1.44-1.58 (m, 2H, H-11’), 1.20-1.34 (1H, m, H-1’) and 1.90 (d, 1H, *J =* 11.4 Hz, H-12’), 2.47 (brs, 1H, H-13’), 1.43-1.56 (m, 1H, H-14’) and 1.56-1.66 (m,1H, H-14’), 1.45-1.58 (m, 1H, H-15’) and 2.37 (d, 1H, *J* = 15.0 Hz, H-15’), 1.69 (s, 3H, H-17’), 1.09 (s, 3H, H-18’), 11.97 (s, 1H, H-19’), 0.89 (s, 3H, H-20’), 8.30 (s,1H, H-1’’), 7.97 (dd,1H, *J =* 8.0; 1.2 Hz, H-4’’), 7.61 (td,1H, *J =* 8.0; 1.2 Hz, H-5’’), 7.51 (td, 1H, *J =* 8.0; 1.2 Hz, H-6’’), 7.94 (dd, 1H, *J* = 8.0, 1.2 Hz, H-7’’); ^13^C-NMR (75.5 MHz, DMSO-*d_6_*) δ_C_: 175.2 (C-3), 40.2 (C-1’), 18.8 (C-2’), 37.6 (C-3’), 44.5 (C-4’), 55.8 (C-5’), 21.9 (C-6’), 41.9, (C-7’), 42.8 (C-8’), 55.2 (C-9’), 39.1 (C-10’), 18.1 (C-11’), 37.5 (C-12’), 46.2 (C-13’), 26.0 (C-14’), 55.5 (C-15’), 63.4 (C-16’), 20.3 (C-17’), 28.6 (C-18’), 178.6 (C-19’), 15.2 (C-20’), 136.3 (C-1’’), 127.3 (C-2’’), 147.9 (C-3’’), 129.2 (C-4’’), 133.0 (C-5’’), 130.4 (C-6’’), 124.1 (C-7’’).

*N1-(E)-[3-Nitrophenyl]-N4-[ent-kauren-16β-methyl-19-oic acid]-thiosemicarbazone* (**11**). Reaction time: 3 h, 72% yield; light yellow amorphous powder; m.p. 217 °C; [α]^20^_D_: -26.54 deg dm^-1 ^g^-1^ cm^3^ (*c* 0.026, DMSO); IR (KBr) υ_max_/cm^-1^: 3,342 (NH), 1,698 (C=O), 1,540-1,505, 836, 736 e 675 (C=C aromatic), 1,352 (N-O), 1,245 (C=S); EI-MS m/z: 526 (M^+•^); ^1^H-NMR δ_H_ (300 MHz, DMSO-*d_6_*): 11.59 (s, 1H, N-2), 7.61 (s, 1H, N-4), 0.74-0.84 (m, 1H, H-1’) and 1.72-1.82 (m, 1H, H-1’), 1.26-1.38 (m, 2H, H-2’), 0.84-1.00 (m, 1H, H-3’) and 2.00 (d, 2H, *J* = 13.5 Hz, H-3’), 1.00 (m, 2H, H-5’ and H-9’), 1.62-1.76 (m, 2H, H-6’), 1.42 (m, 2H, H-7’), 1.48-1.60 (m, 2H, H-11’), 1.26-1.34 (m, 1H, H-12’) and 1.90 (d, 2H, *J =* 11.4 Hz, H-12’), 2.46-2.54 (m, 1H, H-13’), 1.46-1.56 (m, 1H, H-14’) and 1.58-1.60 (m, 1H, H-14’), 1.48-1.59 (1H, m, H-15’) and 2.38 (d, 1H, *J* = 15.0 Hz, H-15’), 1.70 (s, 3H, H-17’), 1.08 (s, 3H, H-18’), 11.95 (s,1H, H-19’), 0.89 (s, 3H, H-20’), 8.43 (s, 1H, H-1’’), 8.14 (s,1H, H-3’’), 8.21 (dd, 1H, *J* = 8.1; 2.4 Hz, H-5’’), 7.70 (t, 1H, *J* 8.1 Hz, H-6’’), 8.15 (dd, 1H, *J* 8.1; 2.4, H-7’’); ^13^C- NMR δ_C_ (75.5 MHz, DMSO-*d_6_*): 175.3 (C-3), 40.2 (C-1’), 18.8 (C-2’), 37.6 (C-3’), 44.5 (C-4’), 55.8 (C-5’), 21.9 (C-6’), 42.0, (C-7’), 42.8 (C-8’), 55.2 (C-9’), 39.1 (C-10’), 18.1 (C-11’), 37.6 (C-12’), 46.1 (C-13’), 26.0 (C-14’), 55.8 (C-15’), 63.4 (C-16’), 20.4 (C-17’), 28.6 (C-18’), 178.6 (C-19’), 15.2 (C-20’), 121.4 (C-1’’), 136.0 (C-2’’), 139.0 (C-3’’), 148.3 (C-4’’), 124.0 (C-5’’), 130.4 (C-6’’), 132.8 (C-7’’).

*N1-(E)-[4-Nitrophenyl]-N4-[ent-kauren-16β-methyl-19-oic acid]-thiosemicarbazone* (**12**). Reaction time: 7 h, 96% yield; yellow amorphous powder; m.p. 219.5 °C; [α]^20^_D_: -5.78 deg dm^-1 ^g^-1^ cm^3^ (*c* 0.026, DMSO); IR (KBr) υ_max_/cm^-1^: 3,338 (NH), 1,697 (C=O), 1,342 (N-O), 1,530-1,490, 842 (C=C aromatic), 1,243 (C=S); EI-MS m/z: 526 (M^+•^); ^1^H-NMR δ_H_ (300 MHz, DMSO-*d_6_*): 11.66 (s,1H, N-2), 7.63 (s,1H, N-4), 0.72-0.82 (m, 1H, H-1’) and 1.70-1.82 (m, 1H, H-1’), 1.22-1.36 (m, 2H, H-2’), 0.90-1.02 (m, 1H, H-3’) and 2.00 (d, 1H, *J =* 12.6 Hz, H-3’), 1.01 (m, 2H, H-5’ and H-9’), 1.59-1.74 (m, 2H, H-6’), 1.41 (m, 2H, H-7’), 1.44-1.58 (m, 2H, H-11’), 1.22-1.34 (m, 1H, H-12’) and 1.91 (d, 1H, *J =* 11.4 Hz, H-12’), 2.40-2.52 (m,1H, H-13’), 1.46-1.56 (m, 1H, H-14’) and 1.58-1.60 (m, 1H, H-14’), 1.44-1.57 (m, 1H, H-15’) and 2.43 (d, 1H, *J* = 14.7 Hz, H-15’), 1.70 (s, 3H, H-17’), 1.09 (s, 3H, H-18’), 11.96 (s,1H, H-19’), 0.89 (s, 3H, H-20’), 8.13 (s, 1H, H-1’’), 7.94 (d, 2H, *J* 9.0 Hz, H-3’’ and H-7’’), 8.24 (d, 2H, *J =* 9.0 Hz, H-4’’ and H6’’); ^13^C-NMR δ_C _(75.5 MHz, DMSO-*d_6_*): 175.3 (C-3), 40.1 (C-1’), 18.8 (C-2’), 37.6 (C-3’ and C-12’), 44.6 (C-4’), 55.8 (C-5’), 21.9 (C-6’), 42.0, (C-7’), 42.8 (C-8’), 55.2 (C-9’), 39.1 (C-10’), 18.1 (C-11’), 46.3 (C-13’), 26.0 (C-14’), 55.5 (C-15’), 63.5 (C-16’), 20.3 (C-17’), 28.6 (C-18’), 178.6 (C-19’), 15.2 (C-20’), 138.8 (C-1’’), 140.5 (C-2’’), 127.9 (C-3’’ and C-7’’), 124.0 (C-4’’ and C-6’’), 147.6 (C-5’’).

*N1-(E)-[2-Chlorophenyl]-N4-[ent-kauren-16β-methyl-19-oic acid]-thiosemicarbazone* (**13**). Reaction time: 2 h, 72% yield; white amorphous powder; m.p. 202.5 °C; [α]^20^_D_: -21.20 deg dm^-1 ^g^-1^ cm^3^ (*c* 0.025, DMSO); IR (KBr) υ_max_/cm^-1^: 3,331 (NH), 1,697 (C=O), 1,535-1,495, 756 (C=C aromatic), 1,246 (C=S), 1,095 (C-Cl); EI-MS m/z: 515 (M^+•^); ^1^H-NMR δ_H_ (300 MHz, DMSO-*d_6_*): 11.58 (s, 1H, N-2), 7.58 (s, 1H, N-4), 0.76-0.82 (m, 1H, H-1’) and 1.70-1.80 (m, 1H, H-1’), 1.22-1.37 (m, 1H, H-2’) and 1.71-1.84 (m, 1H, H-2’), 0.86-0.98 (m,1H, H-3’) and 2.00 (d, 1H, *J =* 12.6 Hz, H-3’), 1.00 (m, 2H, H-5’ and H-9’), 1.58-1.75 (m, 2H, H-6’), 1.28-1.44 (m, 2H, H-7’), 1.43-1.62 (m, 2H, H-11’), 1.20-1.32 (m, 1H, H-12’) and 1.90 (d, 1H, *J =* 11.4 Hz, H-12’), 2.46 (brs, 1H, H-13’), 1.40-1.67 (m, 2H, H-14’), 1.46-1.68 (m,1H, H-15’) and 2.39 (d, 1H, *J* = 14.7 Hz, H-15’), 1.70 (s, 3H, H-17’), 1.09 (s, 3H, H-18’), 11.96 (s, 1H, H-19’), 0.89 (s, 3H, H-20’), 8.44 (s,1H, H-1’’), 7.50 (m, 1H, H-4’’), 7.41 (m, 2H, H-5’’ and H-6’’), 7.97 (m, 1H, H-7’’); ^13^C-NMR δ_C_ (75.5 MHz, DMSO-*d_6_*): 175.2 (C-3), 40.2 (C-1’), 18.8 (C-2’), 37.6 (C-3’ and C-12’), 44.6 (C-4’), 55.8 (C-5’), 21.9 (C-6’), 42.0, (C-7’), 42.8 (C-8’), 55.2 (C-9’), 39.1 (C-10’), 18.1 (C-11’), 46.4 (C-13’), 26.0 (C-14’), 55.6 (C-15’), 63.3 (C-16’), 20.4 (C-17’), 28.6 (C-18’), 178.6 (C-19’), 15.2 (C-20’), 137.5 (C-1’’), 131.4 (C-2’’), 133.0 (C-3’’), 130.0 (C-4’’), 131.2 (C-5’’), 127.7 (C-6’’), 127.2 (C-7’’).

*N1-(E)-[3-Chlorophenyl]-N4-[ent-kauren-16β-methyl-19-oic acid]-thiosemicarbazone* (**14**). Reaction time: 3.5 h, 60% yield; white amorphous powder; m.p. 207 °C; [α]^20^_D_: -8.08 deg dm^-1 ^g^-1^ cm^3^ (*c* 0.026, DMSO); IR (KBr) υ_max_/cm^-1^: 3,338 (NH), 1,697 (C=O), 1,535-1,505, 896, 784 and 683 (C=C aromatic), 1,245 (C=S), 1,075 (C-Cl); EI-MS m/z: 515 (M^+•^); ^1^H-NMR δ_H_ (300 MHz, DMSO-*d_6_*): 11.46 (s, 1H, N-2), 7.56 (s, 1H, N-4), 0.74-0.84 (m,1H, H-1’) and 1.70-1.80 (m,1H, H-1’), 1.26-1.36 (m,1H, H-2’) and 1.72-1.80 (1H, m, H-2’), 0.92-1.00 (m,1H, H-3’) and 2.00 (d, 1H, *J* = 12.6 Hz, H-3’), 1.00 (m, 2H, H-5’ and H-9’), 1.58-1.75 (m, 2H, H-6’), 1.34-1.44 (m, 2H, H-7’), 1.49-1.62 (m, 2H, H-11’), 1.24-1.34 (m,1H, H-12’) and 1.90 (d, 1H, *J* = 11.4 Hz, H-12’), 2.41-2.48 (m,1H, H-13’), 1.44-1.55 (m,1H, H-14’) and 1.60-1.68 (m, 1H, H-14’), 1.48-1.56 (m,1H, H-15’) and 2.42 (d, 1H, *J =* 15.0 Hz, H-15’), 1.70 (s, 3H, H-17’), 1.09 (s, 3H, H-18’), 11.96 (s,1H, H-19’), 0.89 (s, 3H, H-20’), 8.02 (s,1H, H-1’’), 7.45 (m, 2H, H-3’’ and H-6’’), 7.62 (m, 1H, H-5’’), 7.76 (m,1H, H-7’’); ^13^C-NMR δ_C_ (75.5 MHz, DMSO-*d_6_*): 175.2 (C-3), 40.2 (C-1’), 18.8 (C-2’), 37.6 (C-3’ and C-12’), 44.5 (C-4’), 55.8 (C-5’), 21.9 (C-6’), 42.0, (C-7’), 42.8 (C-8’), 55.2 (C-9’), 39.1 (C-10’), 18.1 (C-11’), 37.6 (C-12’), 46.2 (C-13’), 26.0 (C-14’), 55.7 (C-15’), 63.3 (C-16’), 20.4 (C-17’), 28.6 (C-18’), 178.6 (C-19’), 15.2 (C-20’), 139.8 (C-1’’), 136.3 (C-2’’), 129.5 (C-3’’), 133.7 (C-4’’), 125.7 (C-5’’), 130.7 (C-6’’), 126.3 (C-7’’).

*N1-(E)-[4-Chlorophenyl]-N4-[ent-kauren-16β-methyl-19-oic acid]-thiosemicarbazone* (**15**). Reaction time: 2.5 h, 72% yield; white amorphous powder; m.p. 223.5 °C; [α]^20^_D_: -8.00 deg dm^-1 ^g^-1^ cm^3^ (*c* 0.025, DMSO); IR (KBr) υ_max_/cm^-1^: 3,338 (NH), 1,696 (C=O), 1,532, 1,511, 824 (C=C aromatic), 1,245 (C=S), 1,088 (C-Cl); EI-MS m/z: 515 (M^+•^); ^1^H-NMR δ_H_ (300 MHz, DMSO-*d_6_*): 11.43 (s, 1H, N-2), 7.53 (s, 1H, N-4), 0.68-0.78 (m, 1H, H-1’) and 1.68-1.81 (m, 1H, H-1’), 1.24-1.36 (m, 1H, H-2’) and 1.70-1.82 (m,1H, H-2’), 0.80-0.98 (m, 1H, H-3’) and 2.00 (d, 1H, *J* = 12.9 Hz, H-3’), 1.00 (m, 2H, H-5’ and H-9’), 1.60-1.75 (m, 2H, H-6’), 1.30-1.44 (m, 2H, H-7’), 1.46-1.62 (m, 2H, H-11’), 1.20-1.30 (m, 1H, H-12’) and 1.90 (d, 1H, *J* = 11.7 Hz, H-12’), 2.46 (brs, 1H, H-13’), 1.42-1.68 (m, 2H, H-14’), 1.45-1.57 (m, 1H, H-15’) and 2.41 (d, 1H, *J* = 15.0 Hz, H-15’), 1.70 (s, 3H, H-17’), 1.09 (s, 3H, H-18’), 11.96 (s, 1H, H-19’), 0.89 (s, 3H, H-20’), 8.04 (s,1H, H-1’’), 7.70 (d, 2H, *J =* 8.5 Hz, H-3’’ and H-7’’), 7.48 (d, 2H, *J =* 8.5 Hz, H-4’’ and H6’’); ^13^C-NMR δ_C_ (75.5 MHz, DMSO-*d_6_*): 175.1 (C-3), 40.2 (C-1’), 18.8 (C-2’), 37.6 (C-3’ and C-12’), 44.6 (C-4’), 55.8 (C-5’), 21.9 (C-6’), 42.0, (C-7’), 42.8 (C-8’), 55.2 (C-9’), 39.1 (C-10’), 18.1 (C-11’), 46.4 (C-13’), 26.0 (C-14’), 55.6 (C-15’), 63.3 (C-16’), 20.5 (C-17’), 28.6 (C-18’), 178.6 (C-19’), 15.2 (C-20’), 140.1 (C-1’’), 133.0 (C-2’’), 128.9 (C-3’’ and C-7’’), 128.6 (C-4’’ and C-6’’), 134.3 (C-5’’).

### 3.7. Antitrypanosomal assay

Stock solutions of the synthetic compounds and kaurenoic acid (5, 10, 50, and 100 µg/mL) were prepared in dimethylsulfoxide with their final concentrations not exceeding 1.0%. For the assay, epimastigote forms of *T. cruzi* (Y strain) were harvested during the exponential phase of growth, resuspended in liver infusion tryptose broth supplemented with 10% inactivated fetal bovine serum (Gibco Invitrogen Corporation, New York, NY, USA) and plated on 24-well plates at a concentration of 1 × 10^6^ cells/mL. One milliliter of diluted compounds were included in each well and incubated for 96 h at 28 °C. Cell density was determined by counting the parasites in a hemocytometer chamber (Improved Double Neubauer) under a light microscope. All assays were carried out twice, in duplicate, on separate occasions.

### 3.8. Cytotoxicity assay

The cytotoxic effect was evaluated against LLMCK_2_ cells in 96-well plates. A suspension of 2.5 × 10^4^ cells was added to each well and left to grow as a monolayer for 24 h at 37 °C in a 5% CO_2_/air mixture. After this period, different concentrations of the synthetic compounds and kaurenoic acid (10 to 100 µg/mL) were added to the wells, and the plate was incubated for 96 h under the same conditions described above. The cells were fixed in 10% trichloroacetic acid at 4 °C for 1 h, washed five times with distilled water, and allowed to dry at room temperature. A solution of 4% sulforhodamine B (in 1% acetic acid) was added to each well, and the plate was kept protected from light for 30 min at 4 °C. The wells were then washed four times with 1% acetic acid, an aliquot (150 µL) of 10 mM Tris-base was added, and the aliquot was homogenized for 15 min. The absorbance was read at 530 nm in a microplate spectrophotometer, and data were calculated as the percentage of inhibition of growth. A concentration for 50% cellular toxicity (CC_50_) was defined as the concentration that reduced the optic density of treated cells by 50% relative to untreated cells.

## 4. Conclusions

We have synthesized a series of new thiosemicarbazones derived from the natural diterpene kaurenoic acid. These compounds were found to be selective for protozoa and displayed an enhancement of their antitrypanosomal activity when compared to kaurenoic acid (**1**) without significant decreases in cytotoxicity for the tested cells. Nevertheless, the high cytotoxicities expressed by these derivatives could be an indication of their potential as anticancer agents. Further investigations are currently underway to confirm these hypotheses and to elucidate the mode of action and the structure-activity correlations that are involved in antitrypanosomal activity.

## References

[1] de Souza W. (2002). Basic cell biology of Trypanosoma cruzi. Curr. Pharm..

[2] Teixeira A.R.L., Nitz N., Guimaro M.C., Gomes C., Santos-Buch C.A. (2006). Chagas disease. Postgrad. Med. J..

[3] Nwaka S., Ridley R.G. (2003). Virtual drug discovery and development for neglected diseases through public–private partnerships. Nat. Rev. Drug Discov..

[4] Castro J.A., de Mecca M.M., Bartel L.C. (2006). Toxic Side Effects of Drugs Used to Treat Chaga’s Disease (American Trypanosomiasis). Hum. Exp. Toxicol..

[5] Schofield C.J., Jannin J., Salvatella R. (2006). The future of Chagas disease control. Trends Parasitol..

[6] Pink R., Hudson A., Mouriès M.A., Bendig M. (2005). Opportunities and challenges in antiparasitic drug discovery. Nat. Rev. Drug Discov..

[7] Ghisalberti E.L. (1997). The biological activity of naturally occuring kaurane diterpenes. Fitoterapia.

[8] García P.A., de Oliveira A.B., Batista R. (2007). Occurrence, biological activities and synthesis of kaurane diterpenes and their glycosides. Molecules.

[9] Paiva L.A.F., Gurgel L.A., Silva R.M., Tomé A.R., Gramosa N.V., Silveira E.R., Santos F.A., Rao V.S.N. (2003). Anti-inflammatory effect of kaurenoic acid, a diterpene from Copaifera langsdorffii on acetic acid-induced colitis in rats. Vasc. Pharmacol..

[10] Costa-Lotufo L.V., Cunha G.M.A., Farias P.A.M., Viana G.S.B., Cunha K.M.A., Pessoa C., Morais M.O., Silveira E.R., Gramosa N.V., Rao V.S.N. (2002). The cytotoxic and embryotoxic effects of kaurenoic acid, a diterpene isolated from Copaifera langsdorffii oleoresin. Toxicon.

[11] Batista R., Humberto J.L., Chiari E., de Oliveira A.B. (2007). Synthesis and trypanocidal activity of ent-kaurane glycosides. Bioorg. Med. Chem..

[12] Saúde-Guimarães D., Faria A.R. (2007). Substâncias da natureza com atividade anti-*Trypanosoma cruzi*. Rev. Bras. Farmacong..

[13] Vieira H.S., Takahashi J.A., de Oliveira A.B., Chiari E., Boaventura M.A. (2002). Novel derivatives of kaurenoic acid: preparation and evaluation of their trypanocidal activity. J. Braz. Chem. Soc..

[14] Henry G.E., Adams L.S., Rosales J.C., Jacobs H., Heber D., Seeram N.P. (2006). Kaurene diterpenes from Laetia thamnia inhibit the growth of human cancer cells in vitro. Cancer Lett..

[15] Velikova M., Bankova V., Tsvetkova I., Kujumgievi A., Marcucci M.C. (2000). Antibacterial *ent*-kaurene from Brazilian propolis of native stingless bees. Fitoterapia.

[16] Boeck P., Sá M.M., Souza B.S., Cercená R., Escalante A.M., Zachino S.A., Cechinel-Filho V., Yunes R.A. (2005). A simple synthesis of kaurenoic esters and other derivatives and evaluation of their antifungal activity. J. Braz. Chem. Soc..

[17] Bresciani L.F.V., Cechinel-Filho V., Yunes R.A. (2000). Comparative study of different parts of *Wedelia paludosa* by gas chromatography. Nat. Prod. Lett..

[18] Medina J.M., Peixoto J.L.B., Silva A.A., Haraguchi S.K., Falavigna D.L.M., Zamuner M.L.M., Sarragiotto M.H., Vidotti G.J. (2009). Evaluation of the molluscicidal and *Schistosoma mansoni* cercariae activity of *Croton floribundus* extracts and kaurenoic acid. Rev. Bras. Farmacong..

[19] Salatino A., Salatino M.L.F., Negri G. (2007). Traditional uses, chemistry and pharmacology of *Croton* species (Euphorbiaceae). J. Braz. Chem. Soc..

[20] Tenório R.P., Carvalho C.S., Pessanha C.S., de Lima J.G., de Faria A.R., Alves A.J., de Melo E.J.T., Góes A.J.S. (2005). Synthesis of thiosemicarbazone and 4-thiazolidinone derivatives and their in vitro anti-Toxoplasma gondii activity. Bioorg. Med. Chem. Lett..

[21] Beraldo H. (2004). Semicarbazones and thiosemicarbazones: their wide pharmacological profile and clinical applications. Quim. Nova.

[22] Pirrung M.C., Pansare S.V., das Sarma K., Keith K.A., Kern E.R. (2005). Combinatorial optimization of isatin-β-thiosemicarbazones as anti-poxvirus agents. J. Med. Chem..

[23] Hu W.-X., Zhou W., Xia C.-N., Wen X. (2006). Synthesis and anticancer activity of thiosemicarbazones. Bioorg. Med. Chem. Lett..

[24] Kolocouris A., Dimas K., Pannecuoque C., Witvrouw M., Foscolos G.B., Stamatiou G., Fytas G., Zoidis G., Kolocouris N., Andrei G., Snoeck R., de Clercq E. (2002). New 2-(1-adamantylcarbonyl) pyridine and 1-acetyladamantane thiosemicarbazones–thiocarbonohydrazones: cell growth inhibitory, antiviral and antimicrobial activity evaluation. Bioorg. Med. Chem. Lett..

[25] Tarasconi P., Copacchi S., Pelosi G., Cornia M., Albertini R., Bonati A., Dall’Aglio P.P., Lunghi P., Pinelli S. (2000). Synthesis, spectroscopic characterization and biological properties of new natural aldehydes thiosemicarbazones. Bioorg. Med. Chem..

[26] de Oliveira R.B., de Souza-Fagundes E.M., Soares R.P.P., Andrade A.A., Kretti A.U., Zani C.L. (2008). Synthesis and antimalarial activity of semicarbazone and thiosemicarbazone derivatives. Eur. J. Med. Chem..

[27] Pérez-Rebolledo A., Teixeira L.R., Batista A.A., Mangrich A.S., Aguirre G., Cerceretto H., González M., Hernández P., Ferreira A.M., Speziali N.L., Beraldo H. (2008). 4-Nitroacetophenone-derived thiosemicarbazones and their copper (II) complexes with significant in vitro anti-trypanosomal activity. Eur. J. Med. Chem..

[28] Aguirre G., Boiani L., Cerecetto H., Fernández M., González M., Denicola A., Otero L., Gambino D., Rigol C., Olea-Azar C., Faundez M. (2004). *In vitro* activity and mechanism of action against the protozoan parasite Trypanosoma cruzi of 5-nitrofuryl containing thiosemicarbazones. Bioorg. Med. Chem..

[29] Du X., Guoi C., Hansell E., Doyle P.S., Caffrey C.R., Holler T.P., James H., McKerrow J.H., Cohen E. (2002). Synthesis and structure-activity relationship study of potent trypanocidal thio semicarbazone inhibitors of the trypanosomal cysteine protease cruzain. J. Med. Chem..

[30] Fujii N., Mallari J.P., Hansell E., Mackey Z., Doyle P., Zhou Y.M., Gut J., Rosenthal P.J., McKerrow J.H., Guy R.K. (2005). Discovery of potent thiosemicarbazone inhibitors of rhodesain and cruzain. Bioorg. Med. Chem. Lett..

[31] Tenório R.P., Góes A.J.S., de Lima J.G., de Faria A.R., Alves A.J., Aquino T.M. (2005). Thiosemicarbazones: preparation methods, synthetic applications and biological importance. Quim. Nova.

[32] da Silva A.P., Martini M.V., de Oliveira C.M.A., Cunha S., de Carvalho J.E., Ruiz A.L.T.G., da Silva C.C. (2010). Antitumor activity of (-)-α-bisabolol-based thiosemicarbazones against human tumor cell lines. Eur. J. Med. Chem..

[33] Yamaguchi M.U., da Silva A.P.B., Ueda-Nakamura T., Dias-Filho B.P., da Silva C.C., Nakamura C.V. (2009). Effects of a thiosemicarbazide camphene derivative on *Trichophyton mentagrophytes*. Molecules.

[34] da Silva C.C., Almagro V., Marsaioli A.J. (1993). A direct route to terpene isothiocyanates. Tetrahedron Lett..

[35] de Oliveira C.M.A., da Silva C.C., Collins C.H., Marsaioli A.J. (2001). Controlling factors determining the selective HSCN addition to double bonds and their application to the synthesis of 7-isothiocyano-7,8-α-dihydro-bisabolene. J. Braz. Chem. Soc..

